# 4-Meth­oxy-*N*-(4-nitro­benz­yl)aniline

**DOI:** 10.1107/S160053681200846X

**Published:** 2012-03-07

**Authors:** Kamini Kapoor, Vivek K. Gupta, Indresh Kumar, Nisar A. Mir, Rajni Kant

**Affiliations:** a X-ray Crystallography Laboratory, Post-Graduate Department of Physics & Electronics, University of Jammu, Jammu Tawi 180 006, India; b School of Biology & Chemistry, College of Sciences, Shri Mata Vaishno Devi University, Katra 182 320 (J&K), India

## Abstract

In the title compound, C_14_H_14_N_2_O_3_, the nitro group is nearly coplanar with the benzene ring to which it is bonded [dihedral angle = 1.70 (2)°], and this ring is *para*-substituted by the amino­methyl­ene group. The dihedral angle between the benzene rings is 57.8 (1)°. The crystal structure is stabilized by N—H⋯O and C—H⋯O hydrogen bonds and weak C—H⋯π inter­actions are also observed.

## Related literature

For related structures, see: Iwasaki *et al.* (1988 ▶). For the biological properties of aldimines, see: Rjosk & Neumann (1971 ▶); Hillesheim *et al.* (1995 ▶). For bond-length data, see: Allen *et al.* (1987 ▶).
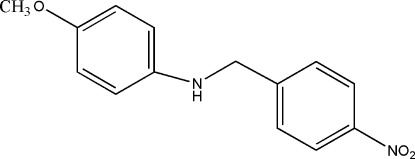


## Experimental

### 

#### Crystal data

C_14_H_14_N_2_O_3_
*M* *_r_* = 258.27
Monoclinic, 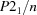

*a* = 7.4993 (3) Å
*b* = 17.1516 (7) Å
*c* = 10.0048 (5) Å
β = 96.861 (4)°
*V* = 1277.65 (10) Å^3^
*Z* = 4
Mo *K*α radiation
μ = 0.10 mm^−1^
*T* = 293 K
0.3 × 0.2 × 0.1 mm

#### Data collection

Oxford Diffraction Xcalibur Sapphire3 diffractometer
Absorption correction: multi-scan (*CrysAlis RED*; Oxford Diffraction, 2010 ▶) *T*_min_ = 0.955, *T*_max_ = 1.000
11435 measured reflections
2511 independent reflections
1692 reflections with *I* > 2σ(*I*)
*R*_int_ = 0.035

#### Refinement

*R*[*F* ^2^ > 2σ(*F* ^2^)] = 0.048
*wR*(*F* ^2^) = 0.130
*S* = 1.05
2511 reflections
178 parameters
H atoms treated by a mixture of independent and constrained refinement
Δρ_max_ = 0.18 e Å^−3^
Δρ_min_ = −0.15 e Å^−3^

### 

Data collection: *CrysAlis PRO* (Oxford Diffraction, 2010 ▶); cell refinement: *CrysAlis PRO*; data reduction: *CrysAlis RED* (Oxford Diffraction, 2010 ▶); program(s) used to solve structure: *SHELXS97* (Sheldrick, 2008 ▶); program(s) used to refine structure: *SHELXL97* (Sheldrick, 2008 ▶); molecular graphics: *ORTEP-3* (Farrugia, 1997 ▶); software used to prepare material for publication: *PLATON* (Spek, 2009 ▶).

## Supplementary Material

Crystal structure: contains datablock(s) I, global. DOI: 10.1107/S160053681200846X/bh2415sup1.cif

Structure factors: contains datablock(s) I. DOI: 10.1107/S160053681200846X/bh2415Isup2.hkl

Supplementary material file. DOI: 10.1107/S160053681200846X/bh2415Isup3.cml

Additional supplementary materials:  crystallographic information; 3D view; checkCIF report

## Figures and Tables

**Table 1:** Hydrogen-bond geometry (Å, °) *Cg*1 and *Cg*2 are the centroids of the nitro­phenyl (C1–C6) and meth­oxy­phenyl (C9–C14) rings, respectively.

*D*—H⋯*A*	*D*—H	H⋯*A*	*D*⋯*A*	*D*—H⋯*A*
N8—H8⋯O1^i^	0.89 (2)	2.42 (3)	3.231 (2)	152.8 (19)
C16—H16*B*⋯O2^ii^	0.96	2.47	3.372 (3)	155
C3—H3⋯*Cg*2^iii^	0.93	2.77	3.560 (2)	143
C6—H6⋯*Cg*2^iv^	0.93	2.87	3.524 (2)	129
C16—H16*A*⋯*Cg*1^v^	0.96	2.96	3.830 (2)	151

Symmetry codes: (i) 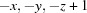; (ii) 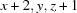; (iii) 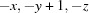; (iv) 
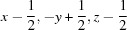
; (v) 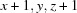.

**Fig. 1.**
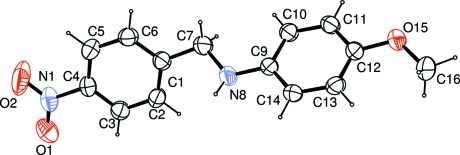
ORTEP view of the molecule with thermal ellipsoids drawn at the 40% probability level. H atoms are shown as small spheres of arbitrary radii.

**Fig. 2.**
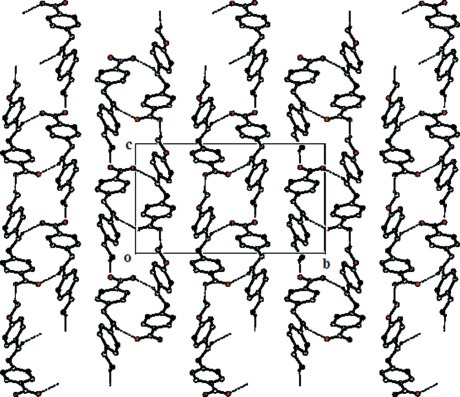
The packing arrangement of molecules viewed down the a axis. The broken lines show the intermolecular N—H···O and C—H···O hydrogen bonds.

## supplementary crystallographic information

### Comment

In continuation of our project on the preparation of various aldimines from *p*-anisidine and aromatic aldehydes in refluxing methanol, the title compound has been prepared using the reductive amination method. In undergoing further applications of aldimines in various cycloaddition reactions (Rjosk & Neumann, 1971; Hillesheim *et al.*, 1995), we observed that aldimines undergo a reductive amination with NaBH_4_ in presence of catalytic amounts of AcOH in MeOH, to afford 4-methoxy-*N*-(4-nitrobenzyl)aniline as one of the products. We further tried to prepare this compound under similar conditions in a separate flask, and the title compound was obtained in high yield (> 90%) through reductive amination of *p*-nitrobenzaldehyde with *p*-anisidine.

The bond lengths in the molecule are within normal ranges (Allen *et al.*, 1987) and comparable with those found in related molecules (Iwasaki *et al.*, 1988). The nitro group is nearly coplanar to the benzene ring to which it is bonded, the dihedral angle being 1.70 (2)°. The 4-methoxy phenyl group is *trans* to the 4-nitro phenyl group about the C7—N8 bond. The torsion angle C1—C7—N8—C9 is 178.22 (17)°. Hydrogen H8 on atom N8 forms an intermolecular hydrogen bond with the nitro O atom O1 of a neighbouring centrosymmetrically related molecule. This interaction links the molecules into N—H···O hydrogen bonded dimers. Dimers are connected *via* C—H···O hydrogen bonds and form chains along the *c*-axis of the unit cell (Table 1, Fig. 2). On the other hand, C—H···π interactions (*Cg*1 is the centroid of the nitro-phenyl ring and *Cg*2 is the centroid of the methoxy-phenyl ring, Table 1) play important role in stabilizing the crystal structure.

### Experimental

To a stirred solution of *p*-nitro-benzaldehyde (0.5 g, 3.3 mmol) in MeOH (10 ml) was added *p*-anisidine (0.41 g, 3.3 mmol) at room temperature and the mixture was refluxed for 4 h. The resulting reaction mixture was cooled to 273 K, which resulted in the precipitation of the corresponding aldimine intermediate. Few drops of AcOH were added, followed by NaBH_4_ (0.09 g, 2.5 mmol), at the same temperature. The combined reaction mixture was stirred additionally for 2 h and quenched with sat. NaHCO_3_ solution, extracted with EtOAc (2×15 ml), and concentrated under reduced pressure. The resulting crude amine compound was crystallized in hexane/EtOAc (2:1), to afford the title compound with 92% yield. ^1^H-NMR: 3.68 (*s*, 3H), 4.38 (*s*, 2H), 6.40 (*d*, 2H), 6.72 (*d*, 2H), 7.65 (*d*, 2H), 8.10 (*d*, 2H).

### Refinement

Hydrogen atom H8 was found in a difference map and refined isotropically. All other H atoms were positioned geometrically and were treated as riding on their parent C atoms, with C—H distances of 0.93–0.97 Å and with *U*_iso_(H) = 1.2*U*_eq_(C) or 1.5*U*_eq_(methyl C).

### Crystal data

**Table:**

C_14_H_14_N_2_O_3_	*F*(000) = 544
*M**_r_* = 258.27	*D*_x_ = 1.343 Mg m^−^^3^
Monoclinic, *P*2_1_/*n*	Mo *K*α radiation, λ = 0.71073 Å
Hall symbol: -P 2yn	Cell parameters from 4747 reflections
*a* = 7.4993 (3) Å	θ = 3.6–29.0°
*b* = 17.1516 (7) Å	µ = 0.10 mm^−^^1^
*c* = 10.0048 (5) Å	*T* = 293 K
β = 96.861 (4)°	Block, red
*V* = 1277.65 (10) Å^3^	0.3 × 0.2 × 0.1 mm
*Z* = 4	

### Data collection

**Table:**

Oxford Diffraction Xcalibur Sapphire3 diffractometer	2511 independent reflections
Radiation source: fine-focus sealed tube	1692 reflections with *I* > 2σ(*I*)
Graphite monochromator	*R*_int_ = 0.035
Detector resolution: 16.1049 pixels mm^-1^	θ_max_ = 26.0°, θ_min_ = 3.6°
ω scans	*h* = −9→9
Absorption correction: multi-scan (*CrysAlis RED*; Oxford Diffraction, 2010)	*k* = −21→21
*T*_min_ = 0.955, *T*_max_ = 1.000	*l* = −12→12
11435 measured reflections	

### Refinement

**Table:**

Refinement on *F*^2^	Secondary atom site location: difference Fourier map
Least-squares matrix: full	Hydrogen site location: inferred from neighbouring sites
*R*[*F*^2^ > 2σ(*F*^2^)] = 0.048	H atoms treated by a mixture of independent and constrained refinement
*wR*(*F*^2^) = 0.130	*w* = 1/[σ^2^(*F*_o_^2^) + (0.0514*P*)^2^ + 0.2077*P*] where *P* = (*F*_o_^2^ + 2*F*_c_^2^)/3
*S* = 1.05	(Δ/σ)_max_ < 0.001
2511 reflections	Δρ_max_ = 0.18 e Å^−^^3^
178 parameters	Δρ_min_ = −0.15 e Å^−^^3^
0 restraints	Extinction correction: *SHELXL97* (Sheldrick, 2008), Fc^*^=kFc[1+0.001xFc^2^λ^3^/sin(2θ)]^-1/4^
0 constraints	Extinction coefficient: 0.0116 (18)
Primary atom site location: structure-invariant direct methods	

### Fractional atomic coordinates and isotropic or equivalent isotropic displacement parameters (Å^2^)

**Table:**

	*x*	*y*	*z*	*U*_iso_*/*U*_eq_	
O1	−0.2703 (2)	0.01096 (11)	0.2223 (2)	0.0948 (6)	
O2	−0.3470 (3)	0.12998 (13)	0.2161 (3)	0.1390 (10)	
N1	−0.2400 (2)	0.07908 (13)	0.24933 (19)	0.0695 (5)	
C1	0.2618 (2)	0.14226 (11)	0.45527 (19)	0.0499 (5)	
C2	0.2220 (3)	0.06469 (12)	0.4248 (2)	0.0581 (5)	
H2	0.3072	0.0265	0.4505	0.070*	
C3	0.0581 (3)	0.04336 (11)	0.3571 (2)	0.0550 (5)	
H3	0.0320	−0.0086	0.3367	0.066*	
C4	−0.0654 (2)	0.10088 (11)	0.32049 (18)	0.0491 (5)	
C5	−0.0304 (3)	0.17839 (12)	0.3476 (2)	0.0563 (5)	
H5	−0.1155	0.2165	0.3210	0.068*	
C6	0.1338 (3)	0.19793 (11)	0.4152 (2)	0.0547 (5)	
H6	0.1593	0.2501	0.4344	0.066*	
C7	0.4411 (3)	0.16668 (12)	0.5260 (2)	0.0636 (6)	
H7A	0.5284	0.1668	0.4619	0.076*	
H7B	0.4319	0.2195	0.5592	0.076*	
N8	0.5039 (2)	0.11659 (10)	0.63639 (18)	0.0597 (5)	
C9	0.6719 (2)	0.13024 (10)	0.71064 (19)	0.0464 (5)	
C10	0.7998 (2)	0.17809 (11)	0.66267 (19)	0.0511 (5)	
H10	0.7719	0.2042	0.5814	0.061*	
C11	0.9671 (3)	0.18720 (11)	0.73402 (19)	0.0531 (5)	
H11	1.0507	0.2194	0.7000	0.064*	
C12	1.0133 (2)	0.14945 (10)	0.85483 (19)	0.0485 (5)	
C13	0.8871 (3)	0.10262 (10)	0.90428 (19)	0.0510 (5)	
H13	0.9152	0.0772	0.9863	0.061*	
C14	0.7188 (2)	0.09308 (10)	0.83278 (19)	0.0505 (5)	
H14	0.6354	0.0610	0.8674	0.061*	
O15	1.18447 (17)	0.16348 (8)	0.91664 (14)	0.0652 (4)	
C16	1.2455 (3)	0.12020 (13)	1.0336 (2)	0.0724 (7)	
H16A	1.1723	0.1321	1.1034	0.109*	
H16B	1.3681	0.1337	1.0633	0.109*	
H16C	1.2375	0.0655	1.0135	0.109*	
H8	0.421 (3)	0.0966 (13)	0.683 (2)	0.072 (7)*	

### Atomic displacement parameters (Å^2^)

**Table:**

	*U*^11^	*U*^22^	*U*^33^	*U*^12^	*U*^13^	*U*^23^
O1	0.0735 (11)	0.0908 (13)	0.1143 (15)	−0.0263 (10)	−0.0125 (10)	−0.0124 (11)
O2	0.0739 (12)	0.1245 (17)	0.199 (3)	0.0363 (13)	−0.0627 (14)	−0.0507 (16)
N1	0.0484 (10)	0.0889 (14)	0.0686 (12)	0.0022 (11)	−0.0032 (9)	−0.0163 (11)
C1	0.0495 (11)	0.0541 (11)	0.0451 (11)	−0.0046 (9)	0.0008 (8)	0.0047 (8)
C2	0.0502 (11)	0.0527 (11)	0.0680 (14)	0.0079 (9)	−0.0063 (9)	0.0017 (10)
C3	0.0534 (11)	0.0500 (11)	0.0604 (13)	−0.0011 (9)	0.0020 (9)	−0.0047 (9)
C4	0.0407 (10)	0.0625 (12)	0.0435 (11)	0.0003 (9)	0.0030 (8)	−0.0043 (9)
C5	0.0539 (12)	0.0570 (12)	0.0570 (12)	0.0117 (10)	0.0021 (9)	0.0001 (10)
C6	0.0603 (12)	0.0471 (11)	0.0557 (12)	−0.0016 (9)	0.0029 (9)	0.0009 (9)
C7	0.0614 (13)	0.0599 (12)	0.0648 (14)	−0.0114 (10)	−0.0119 (10)	0.0118 (10)
N8	0.0488 (10)	0.0669 (11)	0.0601 (11)	−0.0124 (9)	−0.0074 (8)	0.0165 (9)
C9	0.0459 (10)	0.0408 (9)	0.0505 (11)	−0.0027 (8)	−0.0026 (8)	0.0000 (8)
C10	0.0549 (11)	0.0525 (11)	0.0442 (11)	−0.0055 (9)	−0.0011 (9)	0.0067 (9)
C11	0.0520 (11)	0.0520 (11)	0.0545 (12)	−0.0124 (9)	0.0033 (9)	0.0048 (9)
C12	0.0470 (10)	0.0426 (10)	0.0533 (12)	−0.0028 (8)	−0.0044 (9)	−0.0038 (8)
C13	0.0582 (12)	0.0454 (10)	0.0473 (11)	−0.0010 (9)	−0.0020 (9)	0.0067 (8)
C14	0.0503 (11)	0.0457 (10)	0.0544 (12)	−0.0075 (9)	0.0017 (9)	0.0081 (9)
O15	0.0553 (8)	0.0660 (9)	0.0688 (10)	−0.0128 (7)	−0.0150 (7)	0.0086 (7)
C16	0.0653 (13)	0.0741 (14)	0.0711 (15)	−0.0024 (12)	−0.0196 (11)	0.0062 (12)

### Geometric parameters (Å, º)

**Table:**

O1—N1	1.214 (2)	N8—C9	1.404 (2)
O2—N1	1.206 (2)	N8—H8	0.89 (2)
N1—C4	1.462 (2)	C9—C14	1.386 (2)
C1—C6	1.379 (3)	C9—C10	1.390 (3)
C1—C2	1.389 (3)	C10—C11	1.377 (2)
C1—C7	1.502 (2)	C10—H10	0.9300
C2—C3	1.380 (3)	C11—C12	1.378 (3)
C2—H2	0.9300	C11—H11	0.9300
C3—C4	1.373 (3)	C12—C13	1.378 (3)
C3—H3	0.9300	C12—O15	1.378 (2)
C4—C5	1.376 (3)	C13—C14	1.384 (2)
C5—C6	1.373 (3)	C13—H13	0.9300
C5—H5	0.9300	C14—H14	0.9300
C6—H6	0.9300	O15—C16	1.415 (2)
C7—N8	1.433 (2)	C16—H16A	0.9600
C7—H7A	0.9700	C16—H16B	0.9600
C7—H7B	0.9700	C16—H16C	0.9600
			
O2—N1—O1	122.31 (19)	C9—N8—H8	115.1 (14)
O2—N1—C4	118.5 (2)	C7—N8—H8	116.8 (14)
O1—N1—C4	119.17 (19)	C14—C9—C10	117.59 (16)
C6—C1—C2	118.38 (17)	C14—C9—N8	120.47 (16)
C6—C1—C7	119.78 (18)	C10—C9—N8	121.88 (17)
C2—C1—C7	121.81 (17)	C11—C10—C9	120.77 (17)
C3—C2—C1	121.14 (18)	C11—C10—H10	119.6
C3—C2—H2	119.4	C9—C10—H10	119.6
C1—C2—H2	119.4	C10—C11—C12	121.28 (17)
C4—C3—C2	118.24 (18)	C10—C11—H11	119.4
C4—C3—H3	120.9	C12—C11—H11	119.4
C2—C3—H3	120.9	C13—C12—O15	125.72 (17)
C3—C4—C5	122.36 (17)	C13—C12—C11	118.53 (16)
C3—C4—N1	118.84 (18)	O15—C12—C11	115.75 (16)
C5—C4—N1	118.80 (18)	C12—C13—C14	120.43 (17)
C6—C5—C4	118.15 (18)	C12—C13—H13	119.8
C6—C5—H5	120.9	C14—C13—H13	119.8
C4—C5—H5	120.9	C13—C14—C9	121.41 (17)
C5—C6—C1	121.73 (18)	C13—C14—H14	119.3
C5—C6—H6	119.1	C9—C14—H14	119.3
C1—C6—H6	119.1	C12—O15—C16	118.13 (15)
N8—C7—C1	112.89 (16)	O15—C16—H16A	109.5
N8—C7—H7A	109.0	O15—C16—H16B	109.5
C1—C7—H7A	109.0	H16A—C16—H16B	109.5
N8—C7—H7B	109.0	O15—C16—H16C	109.5
C1—C7—H7B	109.0	H16A—C16—H16C	109.5
H7A—C7—H7B	107.8	H16B—C16—H16C	109.5
C9—N8—C7	119.94 (16)		
			
C6—C1—C2—C3	−0.5 (3)	C1—C7—N8—C9	178.22 (17)
C7—C1—C2—C3	−178.52 (19)	C7—N8—C9—C14	166.83 (19)
C1—C2—C3—C4	−0.1 (3)	C7—N8—C9—C10	−16.1 (3)
C2—C3—C4—C5	0.8 (3)	C14—C9—C10—C11	0.7 (3)
C2—C3—C4—N1	−179.66 (18)	N8—C9—C10—C11	−176.45 (18)
O2—N1—C4—C3	−179.1 (2)	C9—C10—C11—C12	−0.1 (3)
O1—N1—C4—C3	−1.3 (3)	C10—C11—C12—C13	−0.7 (3)
O2—N1—C4—C5	0.5 (3)	C10—C11—C12—O15	−179.83 (17)
O1—N1—C4—C5	178.3 (2)	O15—C12—C13—C14	179.92 (17)
C3—C4—C5—C6	−0.8 (3)	C11—C12—C13—C14	0.9 (3)
N1—C4—C5—C6	179.63 (17)	C12—C13—C14—C9	−0.3 (3)
C4—C5—C6—C1	0.1 (3)	C10—C9—C14—C13	−0.5 (3)
C2—C1—C6—C5	0.5 (3)	N8—C9—C14—C13	176.68 (17)
C7—C1—C6—C5	178.53 (18)	C13—C12—O15—C16	7.4 (3)
C6—C1—C7—N8	138.5 (2)	C11—C12—O15—C16	−173.61 (18)
C2—C1—C7—N8	−43.5 (3)		

### Hydrogen-bond geometry (Å, º)

Cg1 and Cg2 are the centroids of the nitrophenyl (C1–C6) and methoxyphenyl (C9–C14) rings, respectively.

**Table:**

*D*—H···*A*	*D*—H	H···*A*	*D*···*A*	*D*—H···*A*
N8—H8···O1^i^	0.89 (2)	2.42 (3)	3.231 (2)	152.8 (19)
C16—H16*B*···O2^ii^	0.96	2.47	3.372 (3)	155
C3—H3···*Cg*2^iii^	0.93	2.77	3.560 (2)	143
C6—H6···*Cg*2^iv^	0.93	2.87	3.524 (2)	129
C16—H16*A*···*Cg*1^v^	0.96	2.96	3.830 (2)	151

Symmetry codes: (i) −*x*, −*y*, −*z*+1; (ii) *x*+2, *y*, *z*+1; (iii) −*x*, −*y*+1, −*z*; (iv) *x*−1/2, −*y*+1/2, *z*−1/2; (v) *x*+1, *y*, *z*+1.
